# Multiple outcomes of the germline p16^INK4a^ mutation affecting senescence and immunity in human skin

**DOI:** 10.1111/acel.14373

**Published:** 2024-10-17

**Authors:** Priya Subramanian, Souraya Sayegh, Phatthamon Laphanuwat, Oliver P. Devine, Carlos Henrique Fantecelle, Justyna Sikora, Emma S. Chambers, Sophia N. Karagiannis, Daniel C. O. Gomes, Anjana Kulkarni, Malcolm H. A. Rustin, Katie E. Lacy, Arne N. Akbar

**Affiliations:** ^1^ Division of Medicine University College London London UK; ^2^ Department of Pharmacology, Faculty of Medicine Khon Kaen University Khon Kaen Thailand; ^3^ Núcleo de Doenças Infecciosas Universidade Federal Do Espírito Santo Vitória Brazil; ^4^ Centre for Immunobiology, Blizard Institute, Barts and the London School of Medicine and Dentistry Queen Mary University of London London UK; ^5^ St. John's Institute for Dermatology, School of Basic and Medical Biosciences, King's College London, Guy's Hospital London UK; ^6^ Breast Cancer Now Research Unit School of Cancer & Pharmaceutical Sciences, Guy's Cancer Centre, King's College London London UK; ^7^ Clinical Genetics Department Guys and St. Thomas' NHS Foundation Trust London UK; ^8^ Department of Dermatology Royal Free Hospital London UK

**Keywords:** cellular senescence, familial melanoma syndrome, immunology, p16, SASP, skin

## Abstract

The integrated behaviour of multiple senescent cell types within a single human tissue leading to the development of malignancy is unclear. Patients with Familial Melanoma Syndrome (FMS) have heterozygous germline defects in the *CDKN2A* gene coding for the cyclin inhibitor p16^INK4a^. Melanocytes within skin biopsies from FMS patients express significantly less p16^INK4a^ but express higher levels of the DNA‐damage protein 𝛾H2AX a than fibroblastic cells. However, patient fibroblasts also exhibit defects since senescent cells do not increase in the skin during ageing and fibroblasts isolated from the skin of patients have increased replicative capacity compared to control fibroblasts in vitro, culminating in abnormal nuclear morphology. Patient derived fibroblasts also secreted less SASP than control cells. Predisposition of FMS patients to melanoma may therefore result from integrated dysregulation of senescence in multiple cell types in vivo. The inherently greater levels of DNA damage and the overdependence of melanocytes on p16 for cell cycle inhibition after DNA damage makes them exquisitely susceptible to malignant transformation. This may be accentuated by senescence‐related defects in fibroblasts, in particular reduced SASP secretion that hinders recruitment of T cells in the steady state and thus reduces cutaneous immunosurveillance in vivo.

## Graphical abstract

(a) In the skin fibroblasts are a key source of SASP production, with melanocytes having significantly less production. In healthy individuals, senescent fibroblasts play a major role in attracting T cells from the circulation by secreting SASP products. Recrutied T cells perform immunosurveillance, eliminating damaged and/malignant cells. (b) In the skin of patients with a *CDKN2A* mutation, multiple senescence‐related defects are present. Both melanocytes and fibroblasts exhibit reduced senescence but are highly susceptible to DNA damage. Senescent fibroblasts secrete reduced levels of SASP products resulting in reduced recruitment of T cells into the skin. This results in reduced immunosurveillance of damaged cells, especially melanocytes that increases the susceptibility of the patients to melanoma.

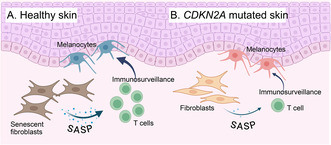

**Abbreviations**
FMS: Familial Melanoma Syndrome
MFI: Mean fluorescence intensity
NHS: National Health Service
PD: Population doubling
SA‐b‐Gal: Senescence‐associated beta galactosidase
SASP: Senescence‐associated secretory phenotype
TAF: Telomere‐associated foci
UCL R&D: University College London Research and Development

## 1 INTRODUCTION

Most of the biology of senescent cells has either been obtained from animal experiments or from experimental cell culture systems in humans (Gorgoulis et al., 2019; Kumari & Jat, 2021). Little is known about how senescent cells behave within human tissues and the impact that defective regulation of senescence has in the development of specific malignancies (Bracken et al., 2024; Laphanuwat et al., 2023). Cellular senescence is the irreversible arrest of cell proliferation in response to potentially oncogenic stress that is established and maintained by at least two crucial contributors to tumour suppression: the p53/p21 and p16^INK4a^/Rb pathways (Campisi, 2013). Characteristic features of senescent cells including enlargement, greater expression of specific proteins such as p16^INK4a^, senescence‐associated beta galactosidase (SA‐β‐Gal), short telomeres, increased p38 expression and the senescence‐associated secretory phenotype (SASP) (Bracken et al., 2024; Campisi, 2013). In addition to excessive proliferation, leading to telomere erosion, senescence‐associated growth arrest can also be induced by ionising radiation, genotoxic drugs and other forms of cellular stress (Burton & Krizhanovsky, 2014; Campisi, 2013; Gorgoulis et al., 2019; Kumari & Jat, 2021). Cellular senescence has been mostly characterised in fibroblasts, but senescent cells of different lineages increase in numerous organs during ageing (Baker et al., 2011; Iske et al., 2024; Krishnamurthy et al., 2006; Ressler et al., 2006; Salminen et al., 2011; Tuttle et al., 2020).

Familial melanoma is a term encompassing all cases of inherited melanoma. It is defined as a family in which 2 first‐degree relatives, or 3 or more members of the same side of the family develop melanoma. *CDKN2A* was the first, and mostly commonly mutated, familial melanoma predisposition gene identified. The *CDKN2A* mutation occurs in approximately 20%–40% of high‐risk families (Rossi et al., 2019), accounting for 1%–2% of melanomas overall, a condition known as the familial melanoma syndrome (FMS) (Potrony et al., 2015). While *CDKN2A gene* knockout in mice is associated with oligozoospermia, propensity to skin tumours including fibrosarcomas and haematological malignancies (Blake et al., 2021) Little is known about FMS, however the recognised clinical phenotype is of multiple melanocytic naevi (usually >50, many of which are atypical), early onset melanoma and pancreatic carcinoma (Mize et al., 2009). The clinical syndrome of a *CDKN2A* mutation resulting in melanomas would suggest that melanocytes are more reliant of the p16/Rb pathway than other cutaneous cell types to become senescent.

In this study we showed multiple senescence‐related changes in melanocytes and in fibroblasts of *CDKN2A*‐deficient (henceforth referred to as FMS) patients. Melanocytes from patients and controls were particularly susceptible to DNA damage in the skin at the steady state but patient melanocytes showed significantly less senescence, suggesting reduced cell cycle control after DNA damage. A key observation was that fibroblasts isolated from FMS patients secreted significantly lower levels of the SASP but have significantly greater replicative capacity than those from healthy controls. Furthermore, patient fibroblasts can proliferate despite harbouring DNA damage. This extends a previous report on cutaneous melanocytes from two patients with mutations in p16^Ink4a^ where these cells showed greater replicative capacity but exhibit chromosomal aberrations at the point of growth arrest (Sviderskaya et al., 2003). Patients also had reduced number of resident memory CD4^+^ and CD8^+^ T cells in the skin suggestive of reduced immune protection. Collectively we have identified multiple cellular senescence associated defects in melanocytes and other cell types in FMS patients in vivo and in isolated cell populations in vitro, that may increase the risk of these patients to the development of melanoma.

## 2 MATERIALS AND METHODS

### 2.1 Recruitment, ethics and exclusion criteria

All biological samples used in this work were collected from either FMS patients (*n* = 16) or healthy age‐ and sex‐matched donors aged 18–85 years (*n* = 13) in line with NHS Research Ethics Committee Queen Square and London‐central (London, United Kingdom) approvals and by the institutional review board (UCL R&D). One–two 5mm cutaneous punch biopsies were obtained from the medial volar forearm, with 50 mL of blood. Exclusion criteria for the controls included history of neoplasm in the last 10 years, immunosuppression, keloid scarring, and vaccination/infective illness within 1 month before sampling. The 16 patients with confirmed germline *CDKN2A* mutation were recruited from a joint Dermatology/Genetics clinic at Guy's Hospital, London. Recruited patients ranged in age from 18 to 68, with a gender split of 13 females and 3 males (Table 1). Despite female preponderance, the *CDKN2A* gene mutation is not known to preferentially affect one gender more than the other (Bishop et al., 2002). The spread of patients in this study is a direct representation of the patients seen in the hospital clinic.

**TABLE 1:** Demographic and mutation data of FMS donors recruited to the study (Chan et al., 2021; Harland et al., 2014; https://clinvarminer.genetics.utah.edu; McKenzie et al., 2010).

Patient	Age	Gender	Protein affected	Variant effect	Functional effect
1	32	F	N/A	N/A	N/A
2	36	F	p16	Insertion	Reduced ability to inhibit S‐phase, reduced phosphorylation of Rb
3	64	F	p16	Deletion/Insertion	Unknown
4	56	F	p16	Missense	Decreased CDK4 binding, reduced structural stability
5	73	F	p16/p14	Missense/Missense	Decreased CDK4/6 binding
6	18	F	p16	Missense	Decreased CDK4/6 binding
7	46	M	p16	Missense	Decreased CDK4/6 binding
8	26	F	p16	Missense	Decreased CDK4/6 binding
9	50	M	p16	Missense	Unknown
10	57	F	N/A	N/A	N/A
11	56	F	p16	Missense	Decreased CDK4/6 binding
12	27	F	p16	Missense	Decreased CDK4 binding
13	44	F	p16	Missense	Decreased CDK4
14	68	M	p16	Missense	Decreased CDK4/6 binding
15	57	F	p16	Missense	Decreased CDK4/6 binding
16	45	F	p16	5'utr	Unknown

Gene mutation loci were retrieved from the medical records of all but two patients (Table S1), whose genetic studies were undertaken in other hospitals whose medical records could not be accessed. Mutations were spread across the gene, and included Exon 1a, Exon 2 and 5'UTR region (Figure S1a). Mutations are known to affect the p16 protein in all patients except one, in which both p16 and p14 are affected (Table 1) (Chan et al., 2021; Harland et al., 2014; McKenzie et al., 2010). Removal of this donor from all analyses did not have any impact on the findings presented. Functional defects resulting from mutation included in 10 patients reduced CDK4 or CDK6 binding, in 1 patient reduced phosphorylation of Rb and an unknown functional outcome in 3 patients (Table 1) (Chan et al., 2021; Harland et al., 2014; McKenzie et al., 2010). We did not observe any association between the location of the gene mutation and the results obtained in this study.

### 2.2 Formalin‐fixation paraffin‐embedded (FFPE) skin biopsies

5mm cutaneous punch biopsies were fixed in 1 mL of 4% paraformaldehyde in PBS, incubated at 4°C for 6 h. Skin biopsies were then loaded into the Leica ASP300S tissue processor (Leica Biosystems, Wetzlar, Germany) for automated paraffin infiltration of tissue, followed by manual paraffin embedding into blocks. Blocks were cut into 3 μm sections and mounted onto polysine‐coated glass slides.

### 2.3 Immunofluorescence

Sections were deparaffinised and antigen retrieval was undertaken with either pH6 or 9 buffer. They were then blocked for 20 min and stained overnight with primary antibodies against p16^INK4a^ (clone 2D9A12, Abcam), Melan A (clone A103, Santa Cruz), CD4 (clone EPR6855, Abcam), CD8 (clone AMC908, ThermoFisher Scientific), FoxP3 (clone PCH101, ThermoFisher Scientific) and CD69 (clone 8B6, ThermoFisher Scientific) followed by secondary antibodies for 60 min. DAPI was applied before imaging using the Leica SPE confocal microscope with LAS X software (Leica Biosystems). High power field (HPF) micrographs were taken at 120× magnification and analysed in Image J. Mean fluorescence intensity (MFI) was calculated by subtracting average background signal from average cellular signal.

A number of other p16 antibody clones were also tested including E6H4 (Cintec), 5A8A4 (Sigma) and polyclonal Ab189302 (Abcam). 2D9A12 was selected based on robust, reproducible staining that increased significantly in healthy human skin during ageing (see below). The further validation of p16^INK4a^ staining for senescent cells was the observation of a strong positive correlation between 2D9A12 antibody staining with telomere‐associated DNA damage foci expression as a second marker of senescence in the skin of healthy volunteers (Figure S1b).

### 2.4 ImmunoFISH for telomere‐associated foci (TAF)

After de‐paraffination and antigen retrieval, sections were blocked for 1 h at room temperature with PBS‐TT containing 8% BSA. Primary antibody solution against 𝛾H2AX and Melan A antibody was applied for overnight incubation, followed by secondary antibody solution for 60 min. 4% paraformaldehyde in PBS was used for cross‐linking with subsequent sequential treatment with 70%, 90% and 100% −20°C ethanol for 2 min each. Hybridisation mix including Tris, magnesium chloride, deionised formamide, water, blocking buffer, and PNA probe (Panagene, TelC Cy3) was applied. DNA was denatured for 10 min at 82°C and incubated for 2 h for hybridisation. Sections were washed once for 10 min in 70% formamide/30% saline sodium citrate (SSC) buffer, and twice for 10 min each in SSC buffer. DAPI was applied along with coverslips. Slides were imaged using Leica SPE confocal microscope with LAS X software (Leica Biosystems). High power field (HPF) micrographs were taken at 120× magnification and analysed manually for colocalising telomere and 𝛾H2AX signals using Image J. MFI was calculated by subtracting average background signal from average cellular signal.

### 2.5 Isolation and culture of cutaneous melanocytes and fibroblasts

5mm cutaneous punch biopsies with incubated overnight with TrypLE (Gibco 12563011). Dermis and epidermis were separated, with the epidermis mechanically disaggregated and plated in complete melanocyte media (Lonza CC3249, CC4510 and CC3250) in one well of a 6 well plate for culture. The dermis was digested using the Miltenyi Whole Skin Dissociation Kit protocol (Miltenyi 130‐101‐540).

### 2.6 Senescence induction

Senescence was induced in P4‐7 primary fibroblasts by exposing them to either mitomycin‐C 0.2 μm in cell culture media for 48 h or 10 Gy x‐ray irradiation at a dose of 5 Gy/min.

Cells were then left for 10–14 days in culture for senescence to develop. Senescence was confirmed using an SA‐β‐gal assay (Cell Signalling). For replicative senescence, fibroblasts were serially passaged until morphologically large with a static cell population.

Population doublings were calculated using the formula PD = log10 (total no./start no.)/log2 as described previously (Plunkett et al., 2005). Senescence in P4‐6 melanocytes was achieved using mitomycin C 0.2 μM in cell culture media for 48 h.

### 2.7 Immunocytochemistry

Cells were plated in a 96‐well Optical Bottom plate at a density of 5000 cells/well and incubated at 37°C, 5% CO_2_ for 24 h. Cells were fixed in situ with 4% PFA in PBS for 10 min at room temperature followed by permeabilization with 0.1% PBS‐Triton for 10 min at room temperature. Cells were then blocked for 20 min at room temperature with Dako Protein Block (Agilent X090930‐2), before application of primary antibody cocktail overnight against Ki67 (clone B126.1, Abcam), 𝛾H2AX (Ser139) (clone 20E3, Cell Signalling), p16^INK4a^ (clone EPR1473, Abcam) and p21 (clone EPR362, Abcam) followed by a secondary antibody cocktail with 60 min incubation. Cells were then incubated with Phalloidin stain for 20 min followed by DAPI. The plate was imaged using the Cell Discoverer 7 microscope (Zeiss) and images were analysed using the Zeiss Zen Software.

For SA‐β‐gal, fibroblasts were plated in a 6‐well plate at a density of 3 × 10^4^ cells/well, fixed and stained for SA‐β‐gal using the Senescence β‐Galactosidase Staining Kit and protocol (Cell Signalling #9860). After overnight incubation any crystal precipitates were dissolved with DMSO, and the plate imaged with the Zeiss Primovert microscope. Image analysis was conducted with Zeiss software.

### 2.8 Cytometric bead array

Non‐senescent and senescent fibroblasts were seeded into a 6‐well plate (at 16 × 10^4^ cells/well) for cytokine quantification. Culture supernatants were collected after 36 h and assessed using cytometric bead array (BD Biosciences) for concentrations of CXCL10, CCL5, CCL2, IL‐6 and IL‐8 according to the manufacturer's instructions. Data was acquired using the BD Verse Flow Cytometer (BD Biosciences) and analysed with FCAP Array v3 software (BD Biosciences).

### 2.9 Western blot

1 × 10^6^ fibroblast cell pellets were lysed with RIPA buffer (Sigma‐Aldrich) protease inhibitor (Cell Signalling) cocktail for 30 min on ice. Lysate protein concentration was determined using the Pierce™ bicinchoninic acid (BCA) assay kit (ThermoFisher Scientific). Lysate containing 30 μg protein was added to a cocktail of NuPAGE® LDS Sample Buffer, reducing agent and dye, with heating to 95°C for 5 min. Proteins were separated by electrophoresis using Bis‐Tris 10% Precast 10‐well Gel (Life Technologies) at 100 V for 1.5–2 h, then transferred onto a PVDF membrane (Amersham Hypond‐P, GE Healthcare) at 100V for 2 h. The membranes were blocked in 5% BSA in TBT‐T and incubated overnight with primary antibodies against p16^INK4a^ (clone EPR1473, Abcam), p21 (clone EPR362, Abcam), and GAPDH (14C10, Cell Signalling) followed by HRP‐conjugated secondary antibodies (Cell Signalling). ECL Prime Detection Reagent (Merck Life Science) was added and protein bands imaged using the Syngene G:Box (Syngene). Membranes were stripped using Restore Stripping Buffer (ThermoFisher Scientific) followed by re‐probing. Quantification of protein bands was undertaken in Image J, with normalisation to expression of GAPDH.

### 2.10 Flow cytometry

Peripheral blood mononuclear cells were isolated from blood using Ficoll‐Paque (Amersham Biosciences) density centrifugation. Surface staining was undertaken with fluorochromeconjugated antibody cocktail against CD3‐BV500 (clone UCHT1, BD Biosciences), CD4BUV395 (clone SK3, BD Biosciences), CD8‐BUV737 (clone SK1, BD Biosciences), CLA‐FITC (clone HECA‐452, BioLegend) and CCR4‐BV605 (clone IGI, BD Biosciences) at appropriate concentrations on ice for 45 min. Samples were acquired using the LSR‐II flow cytometer (BD Biosciences) and analysed using FlowJo software.

### 2.11 Statistical analyses

Statistical analyses were undertaken in GraphPad Prism software version 10. Distribution of data was determined using normality tests. Normal/Gaussian distributed data was analysed with the unpaired Student's T‐test, and the Whitney‐Mann test for non‐parametric data. Oneway ANOVA was conducted for analysis of more than two datasets. Statistical comparisons of age‐matched growth curves were performed in R version 4.2.2 and the statmod package using the CGGC permutational test (Elso et al., 2004; R Core Team, 2022). *p* ≤ 0.05 was considered statistically significant.

## 3 RESULTS

### 3.1 Reduced p16^INK4a^ expression in *CDKN2A* ‐ deficient skin

**FIGURE 1:**
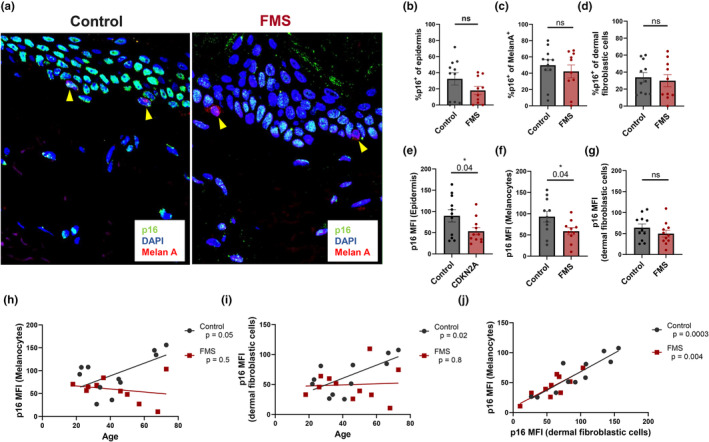
Decreased p16^INK4a^ expression by melanocytes in FMS patient skin. Formalin‐fixed‐paraffin‐embedded (FFPE) 5 mm skin biopsies from FMS patients (*n* = 10–11) and healthy, matched control donors (*n* = 11) were stained by immunofluorescence for p16^INK4a^ (green), Melan A (red) with DAPI nuclear counterstain (blue). Representative p16^INK4a^ staining shown in control and FMS patient skin (a) Yellow arrowheads indicate melanocytes. Graphs show (b) Average percentage of p16^INK4a+^ keratinocytes; (c) Average percentage of p16^INK4a+^ melanocytes; (d) Average percentage of p16^INK4a+^ dermal fibroblastic cells; (e) Average epidermal cellular p16^INK4a^ MFI; (f) Average melanocyte p16^INK4a^ MFI; (g) Average dermal fibroblastic cell p16^INK4a^ MFI; (h) Correlation between p16^INK4a^ MFI and age in melanocytes; (i) Correlation between p16^INK4a^ MFI and age in dermal fibroblastic cells; (j) Correlation between melanocyte and dermal fibroblastic cell p16^INK4a^ MFI. Data are represented as mean ± SEM. Statistical significance calculated by unpaired two‐tailed Student's *t*‐test. ns, *p* > 0.05; *p ≤ 0.05.

The effect of the *CDKN2A* mutation was initially assessed by studying its protein product, p16^INK4a^, in normal human skin by immunofluorescence. The proportion of p16^INK4a+^ cells in epidermal cells, melanocytes and dermal fibroblastic cells was not significantly altered in control compared to FMS patient skin (Figure 1a–d). No age‐related changes were apparent in proportions of p16^INK4a+^ epidermal cells, dermal fibroblastic cells and melanocytes in the skin of patients and controls (Figure S2a–c). However, the level of p16^INK4a^ protein expression was significantly decreased in epidermal cells and melanocytes of FMS patients compared to healthy controls (Figure 1e,f). Therefore, the level of expression of p16^INK4a^ in melanocytes, but not the proportion of cells that expressed this molecule, was altered in the skin of FMS patients. There was a trend towards a reduction of p16^INK4a^ expression in dermal fibroblastic cells, but this was not significant (Figure 1g). We did not observe any significant gender‐related influences in p16^INK4a^ expression in the skin of both controls and FMS patients in our cohort that was predominantly female (Figure S3a–f).

We observed an age associated increase of p16^INK4a^ expression in melanocytes and dermal fibroblastic cells in the skin of healthy controls but not in FMS patients (Figure 1h,i). This indicated a defect in p16^INK4a^ expression in both cell types during ageing. We also showed that the level of p16^INK4a^ expression in melanocytes and dermal fibroblastic cells in the same donors was strongly correlated with each other in both healthy control and FMS patient skin (Figure 1j). Suggesting that p16^INK4a^ expression is co‐regulated across different cell types in the same individuals.

### 3.2 Reduced senescence by assessing telomere‐associated DNA damage foci (TAF ^+^) in FMS patient melanocytes

**FIGURE 2:**
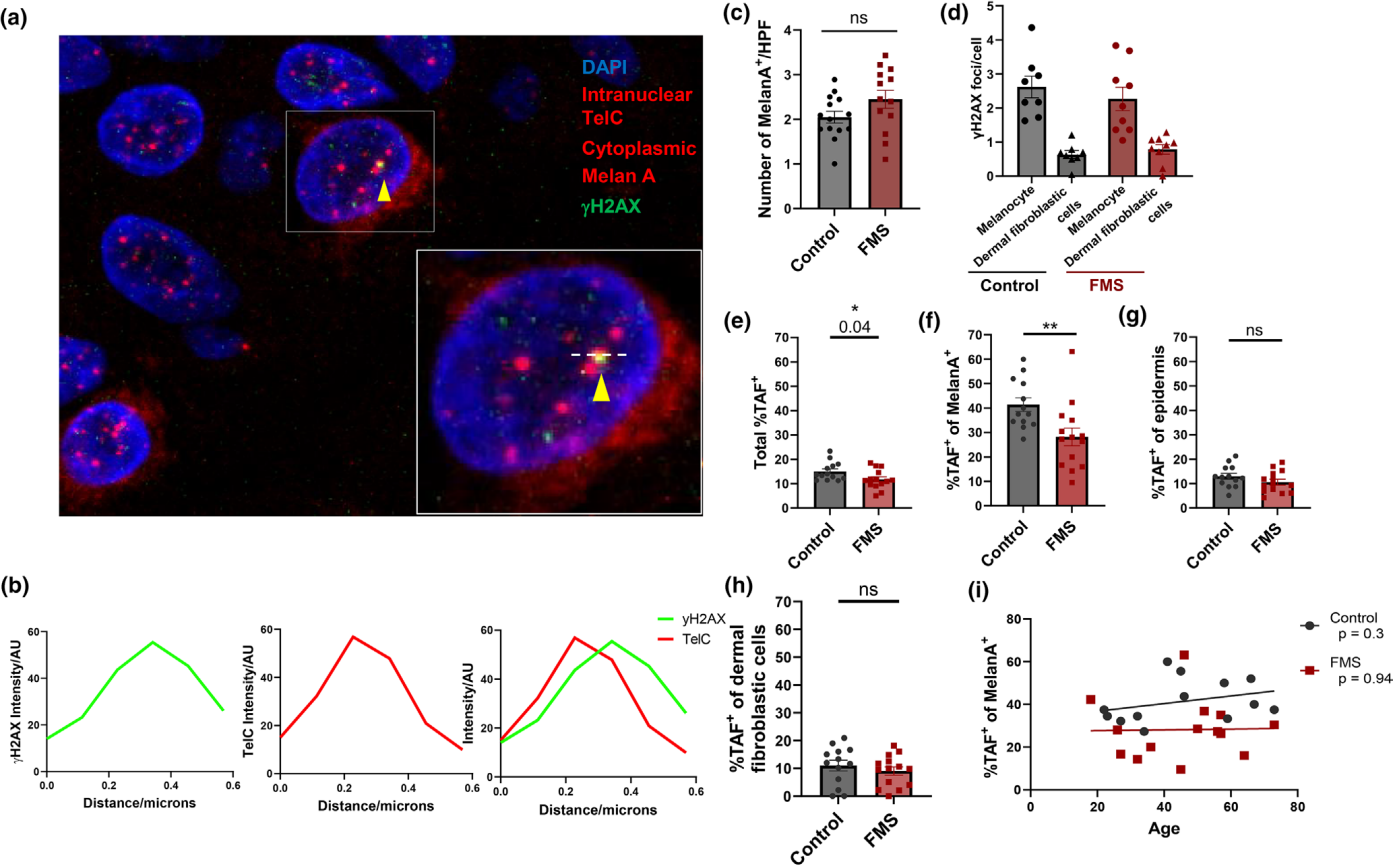
Fewer TAF^+^ cutaneous melanocytes in FMS patients. Formalin‐fixed‐paraffin‐embedded (FFPE) 5 mm skin biopsies from FMS patients (*n* = 13–14) and healthy, matched control donors (*n* = 13–14) were cut into 3 μm sections and stained by immunoFISH for TAF and Melan A. (a) Representative high‐power confocal Z‐stack image of epidermis and superficial dermis showing telomeres (red intranuclear), Melan A (red membrane), 𝛾H2AX (green) with nuclear DAPI counterstain (blue). All Melan A+ cells from one skin section were imaged for each donor (20–30 cells per donor). Yellow arrowhead indicates colocalising TelC and 𝛾H2AX amounting to TAF. Inset white box shows a magnified TAF+ melanocyte, the dashed white line passing through a TAF is represented by histograms (b) showing the relative intensity of 𝛾H2AX (green line), Tel C (red line) across the distance of the white line. Graphs show (c) Mean number of Melan A^+^ cells/high power field; (d) FMS (*n* = 8), control donors (*n* = 9) mean numbers of nuclear 𝛾H2AX foci; (e) Percentage of cutaneous TAF^+^ cells; (f) Percentage of TAF^+^ melanocytes; (g) Percentage of TAF^+^ epidermal cells; (h) Percentage of TAF^+^ dermal fibroblastic cells; (i) Correlation between TAF^+^ melanocytes and age. Data are represented as mean ± SEM. ns, *p* > 0.05; *p ≤ 0.05; **p ≤ 0.01.

It was not clear if the decreased p16^INK4a^ expression in melanocytes of FMS patients indicated decreased senescence of these cells. Skin sections from healthy controls and FMS patients were stained for the DNA‐damage associated protein 𝛾H2AX, telomeres and melanocytespecific marker Melan A using immunoFISH. Co‐localisation of 𝛾H2AX and telomeres, known as telomere‐associated DNA damage foci (TAF), was utilised as an alternative method for identifying senescent cells (Figure 2a,b).

There were comparable numbers of melanocytes in FMS patient and healthy control skin samples (Figure 2c). Melanocytes in both control and FMS skin have more DNA damage than dermal fibroblastic cells (Figure 2d). The total proportion of senescent (TAF^+^) cells across the skin was reduced in FMS donors when compared to healthy controls (Figure 2e). This is accounted for by a reduction in the proportion of TAF^+^ FMS patient melanocytes compared to those from healthy controls m (Figure 2f) but not in epidermal cells and dermal fibroblastic cells (Figure 2g,h). There were fewer senescent (TAF^+^) melanocytes in FMS patient skin across all age groups compared to healthy controls (Figure 2i). However, we did not observe any differences in senescent epidermal cells, dermal fibroblastic cells and melanocytes between all age groups in both patients and controls (Figure S4).

We also investigated other markers of senescence in the skin of the healthy controls and FMS patients. The reduced nuclear expression of High mobility box group 1 protein (HMGB1) has been associated with senescent melanocytes in skin (Victorelli et al., 2019). We identified HMGB1 staining in both patients and the controls (Figure S5a,b). When we combined the data of patient and controls we found a significant age‐related decrease in nuclear HMGB1 expression in total skin and also in melanocytes with a similar trend found when both patients and control were investigated separately (Figure S5c,d).

However there were no significant differences when we compared the level of expression of HMGB1 in total skin, epidermis, melanocytes or dermal fibroblastic cells between controls and FMS patients (Figure S5e–h).

The decrease of lamin B1 has been validated as a marker for cellular senescence in vitro and in vivo (Ivanov et al., 2013). We observed reproducible Lamin B1 staining in the skin of both control and FMS patients but did not observe Lamin B1 staining in any melanocytes in both groups (Figure S5i, representative image from 9 controls and 9 FMS patients).

A characteristic of senescent cells is their lack of proliferative activity (Hayflick, 1965). We investigated whether the level of proliferation of different cell types was altered in the skin of FMS patients compared to controls. Using ki67 as a proliferative marker we found that a few cells in the lower epidermis of patients and controls, consistent with the basal keratinocyte stem cell layer expressed ki67 in both groups (Figure S5j, representative image from 9 controls and 9 patients). The level of ki67 expression was not different in the skin of controls and FMS patients and we did not identify any melanocytes that expressed ki67 in both groups.

### 3.3 FMS patient fibroblasts become senescent using the p21 pathway

Low cell numbers restricted the extensive use of isolated melanocytes in in vitro experiments. We also found that senescent melanocytes secreted significantly less SASP that senescent fibroblasts from the same individuals (Figure S5). We therefore focussed on dermal fibroblasts from control and FMS patients as these cells, like melanocytes, exhibited chromosomal aberrations at the point of growth arrest (Sviderskaya et al., 2003) and ageassociated dysfunctional regulation of senescence (Figure 1h,i). Primary dermal fibroblasts were isolated from the skin of healthy controls and age‐ and sex‐matched FMS patients then cultured and induced to become senescent by either mitomycin C treatment or x‐ray irradiation. We found that isolated dermal fibroblasts could be induced to become senescent by either treatment as confirmed by SA‐β‐Gal staining (Figure S6a,b).

**FIGURE 3:**
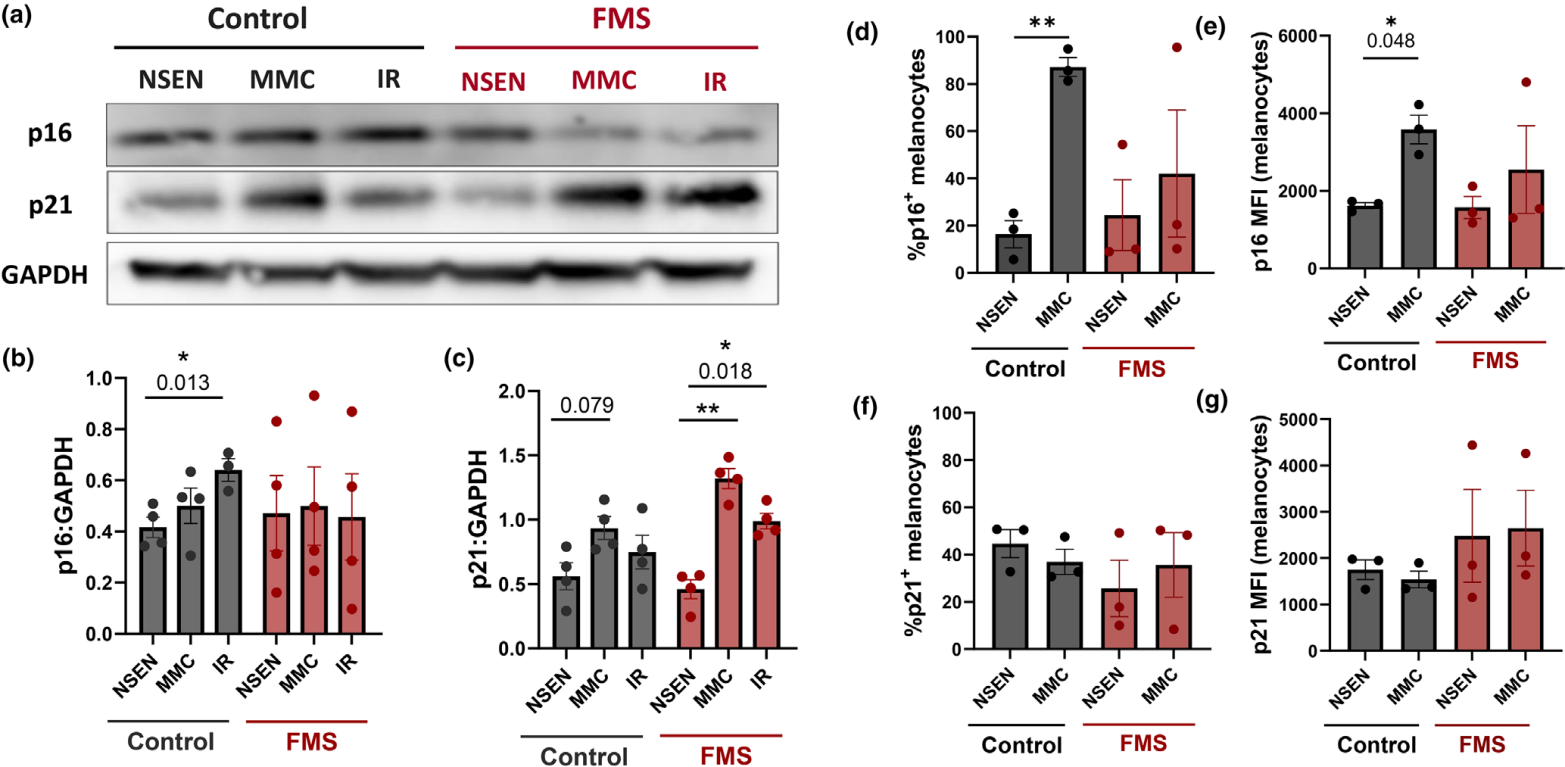
FMS fibroblasts upregulate p21 instead of p16^INK4a^ to become senescent, FMS melanocytes upregulate neither. (a–c) Cutaneous fibroblasts were isolated from healthy donor (*n* = 3–4) and FMS donor (*n* = 4) skin. They were cultured either in their non‐senescent state (NSEN) or induced to senescence with mitomycin C (MMC) or x‐ray irradiation (IR). 15 days after senescence‐induction, all cells were lysed and protein extracted for Western Blot. (a) Representative immunoblots. Graphs show mean grey value of detected protein normalized to GAPDH (B) p16^INK4a^; (c) p21. (d–g) Cutaneous melanocytes were isolated from healthy donors (*n* = 3) and FMS donors (*n* = 3) skin. They were cultured either in their non‐senescent state (NSEN) or induced to senescence with mitomycin C (MMC). 15 days after senescence‐induction, all cells were fixed and stained for immunocytochemistry. Graphs show (d) Percentage of p16^INK4a+^ melanocytes; (e) p16^INK4a^ MFI; (f) Percentage of p21^+^ melanocytes; (g) p21 MFI. Data are represented as mean ± SEM. Statistical significance calculated by Sidak's multiple comparisons test. *p ≤ 0.05; **p ≤ 0.01.

We next investigated which senescence signalling pathways were being engaged in dermal fibroblasts during the induction of senescence. Senescence induction in healthy fibroblasts by irradiation significantly induced p16^INK4a^ expression. However, this did not occur in senescent FMS patient fibroblasts (Figure 3a,b). While senescent fibroblasts from controls and patients could upregulate p21, the increase was only significant in the patient cells (Figure 3a,c). This suggests that fibroblasts can activate both p16^INK4a^ and p21 to become senescent. However, FMS patient fibroblasts that have deficient p16^INK4a^ may rely on p21 to become senescent after irradiation or mitomycin C treatment.

We also investigated the regulation of senescence in melanocytes after mitomycin C treatment using immunocytochemistry to accommodate low cell numbers. Mitomycin C‐treated melanocytes from healthy controls showed significantly increased p16^INK4a^ but not p21 expression (percentage of positive cells and MFI; Figure 3d–g). Mitomycin C‐treated FMS patient melanocytes did not upregulate p16^INK4a^ or p21 (Figure 3d–g). This suggests that melanocytes are more dependent on p16^INK4a^ than fibroblasts to become senescent, confirming previous reports (Sviderskaya et al., 2002). This also provides a reason for the preferential reduction of senescence in melanocytes compared to other cell types in the skin of FMS patients (Figure 2f).

### 3.4 *CDKN2A‐*mutation attenuates fibroblast inflammatory protein secretion

**FIGURE 4:**
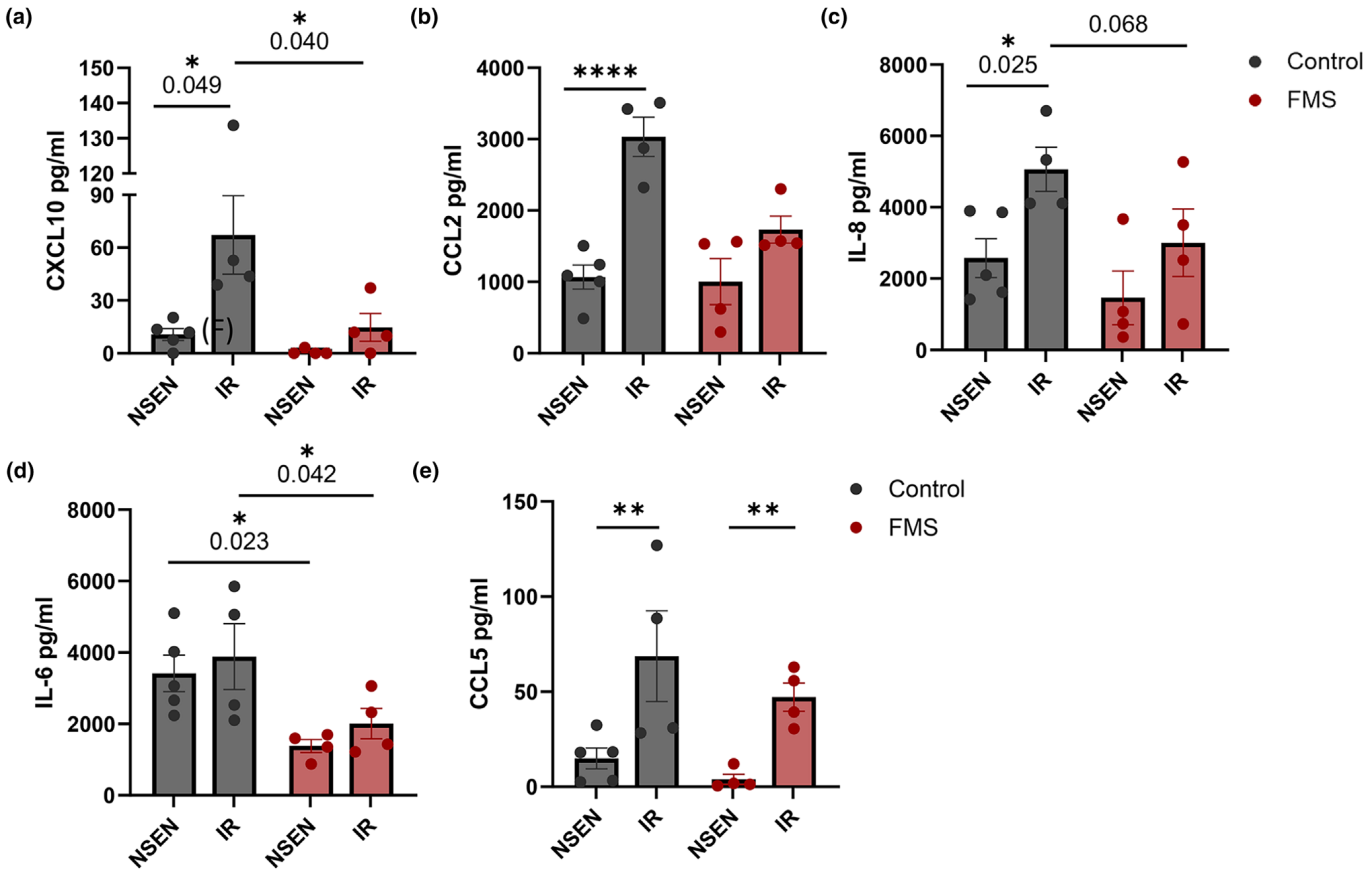
*CDKN2A‐*mutation attenuates fibroblast inflammatory protein secretion. Supernatants from healthy donor (*n* = 4–5) and FMS patients (*n* = 4) non‐senescent (NSEN) and senescent (IR) fibroblasts were cultured for 24 h and analysed for cytokine content using cytometric bead array. Graphs show concentrations of cytokines in supernatants as follows: (a) CXCL10; (b) CCL2; (c) CCL5; (d) IL‐8; (e) IL‐6; Data are represented as mean ± SEM. Statistical significance calculated by Dunn's multiple comparisons test, ns, *p* > 0.05; *p ≤ 0.05; **p ≤ 0.01; ***p ≤ 0.0001.

Previous studies have shown that knockdown of p16^INK4a^ in cell lines (Buj et al., 2021) and mouse models of intervertebral disc degeneration (Che et al., 2020) decreases expression of SASP factors and inflammatory proteins. We next investigated the functional properties of senescent fibroblasts and melanocytes from healthy controls and FMS patients. Senescent cells secrete a wide range of bioactive mediators that are involved with immune recruitment, tissue homeostasis and repair (Coppé et al., 2010; Kumari & Jat, 2021). Fibroblasts and melanocytes from control skin were isolated and induced to senescence with x‐ray irradiation. Supernatants were collected and analysed using cytometric bead arrays for CCL5, CXCL10, CCL2, IL‐6 and IL‐8. These chemokines were uniquely selected in view of their importance in lymphocyte recruitment (Harlin et al., 2009), their role in host response against cancer, and the contribution of IL‐6 and IL‐8 as prototypic senescence‐associated secretory phenotype (SASP) components (Kumari & Jat, 2021). In healthy controls, senescent fibroblasts secrete significantly more CCL5, CXCL10, CCL2 and IL‐8 than senescent melanocytes (Figure S5a–d). Furthermore, fibroblasts from FMS patients unlike those from healthy controls failed to significantly upregulate CXCL10, CCL2 and IL‐8 after induction of senescence by irradiation (Figure 4a–c). Senescent patient fibroblasts also had reduced CXCL10 and IL‐6 production as compared to senescent healthy control fibroblasts (Figure 4a,d). The senescent patient fibroblasts do however upregulate production of CCL5 significantly compared to non‐senescent cells (Figure 4e), showing not all cytokines are similarly affected by defective p16 expression. Interestingly neither healthy control nor FMS patient fibroblasts significantly upregulate IL‐6 production after induction of senescence nevertheless patient fibroblasts secrete significantly less IL‐6 than healthy control cells overall (Figure 4d).

### 3.5 Increased replicative capacity leading to nuclear abnormalities in FMS patient fibroblasts

**FIGURE 5:**
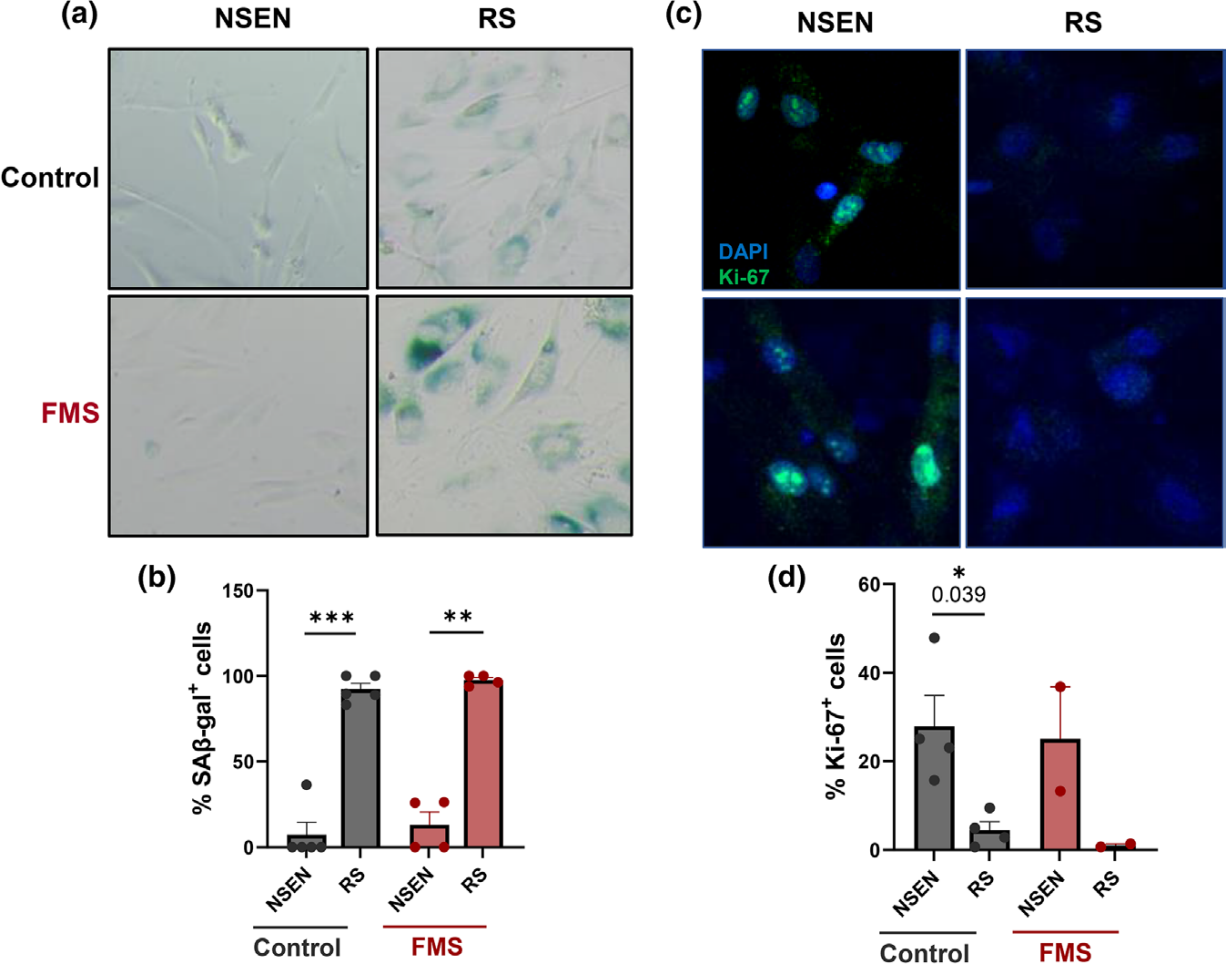
Confirmation of replicative senescence in control and FMS patient fibroblasts. Cutaneous fibroblasts were isolated from healthy donor (*n* = 4–5) and FMS patients (*n* = 2–4) skin. They were serially passaged over an extended period until replicative senescence (RS) was achieved. (a) Representative images of SAβ‐Gal (blue) and (b) Ki67 staining (green). Graphs showing (c) Percentage of SAβ‐Gal+ cells; (d) Percentage of Ki67+ cells; Data are represented as mean ± SEM. Statistical significance calculated by Sidak's multiple comparisons test. *p ≤ 0.05; **p ≤ 0.01; ***p ≤ 0.001.

**FIGURE 6:**
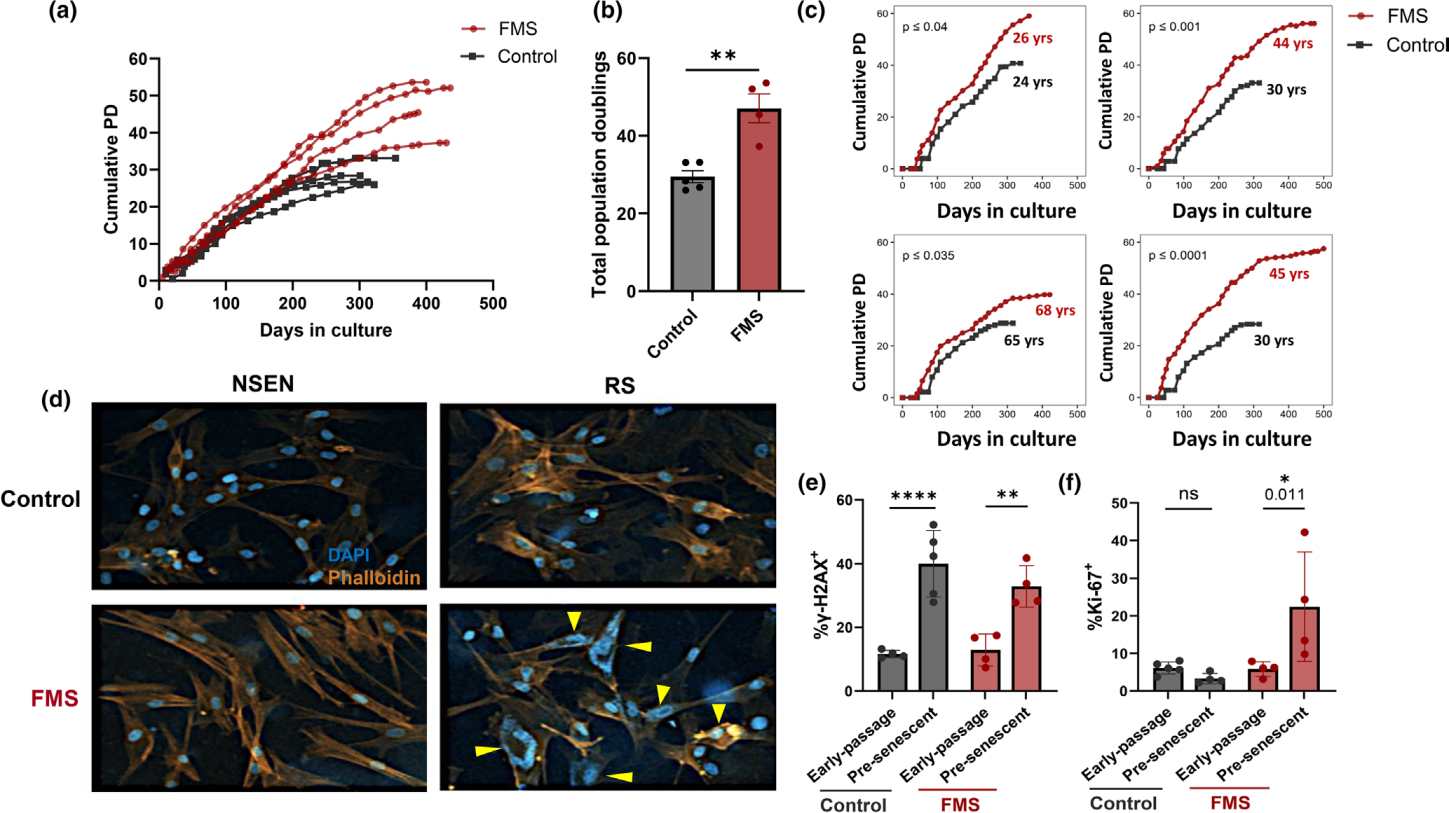
Increased replicative capacity of FMS patient fibroblasts. Cutaneous fibroblasts were isolated from healthy donors (*n* = 5; ages 24, 30, 30, 65 and 66 years old) and FMS patients (*n* = 4; ages 26, 44, 45 and 68 years old) skin. They were serially passaged over an extended period until replicative senescence was achieved. (a) Graph showing cumulative population doublings over time; (b) Total population doublings per donor. (c) Pairwise comparisons of growth curves, the ages of the patients and controls are indicated in each panel and they were also gender matched. (d) Representative images showing morphological changes of senescent fibroblasts using DAPI nuclear stain (blue) and phalloidin actin stain (orange). Graphs showing (e) Percentage of 𝛾H2AX^+^ cells and (f) Percentage of Ki67^+^ cells in early‐passage and pre‐senescent fibroblasts. Data are represented as mean ± SEM. Statistical significance for (b) unpaired two‐tailed Student's *t*‐test, (c) permutation tests, (e, f) Sidak's multiple comparisons test. *p ≤ 0.05; **p ≤ 0.01; ****p ≤ 0.0001.

To assess the replicative capacity of *CDKN2A*‐deficent patient fibroblasts we serially passaged primary dermal fibroblasts from patients and healthy controls until replicative senescence was reached. Replicative senescence could be achieved in both control and patient cells that was confirmed by increased SA‐β‐Gal (Figure 5a,b) and reduced Ki67 (Figure 5c,d). We calculated the replicative capacity of control and patient cells using the standard formula for cumulative population doubling as described in the Materials and Methods. We found that FMS patient fibroblasts from all donors achieved significantly more cumulative population doublings and delayed replicative senescence compared to age matched healthy controls (Figure 6a,b). FMS patients and control donors were compared directly, being matched based on gender and closest age‐match. This revealed a statistically significant increase in the expansion of patient cells compared to those from healthy age and sex matched controls in four separate experiments (Figure 6c).

Morphologically, senescent fibroblasts from all donors were larger compared to nonsenescent counterparts. However nuclear abnormalities were present in senescent FMS fibroblasts, consisting of larger, irregular and/or fragmented nuclear structures (Figure 6d), implying abnormality of the DNA/chromosomal structure. Furthermore when investigating presenescent fibroblasts (i.e. two passages before replicative senescence), cells from both controls and patients showed a significant increase in the DNA damage protein 𝛾H2AX (Figure 6e) but FMS patients fibroblasts were still able to proliferate. This indicated a deficiency of senescence‐associated cell cycle control as shown by the increase Ki‐67^+^ cells (Figure 6f).

### 3.6 Decreased CD8 ^+^ and CD4 ^+^ T cell numbers in FMS patient skin

**FIGURE 7:**
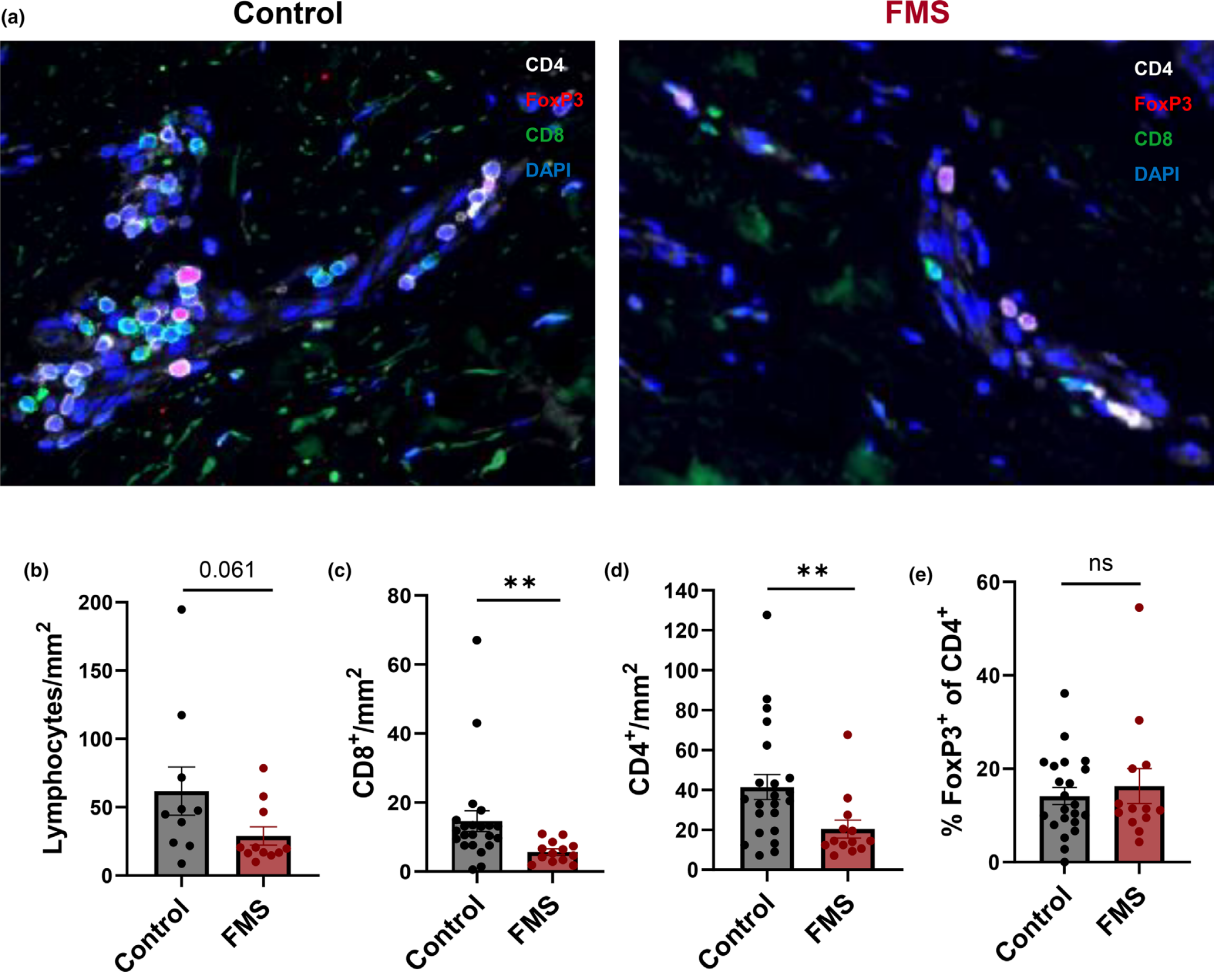
Reduced numbers of CD8^+^ and CD4^+^ T cells in FMS patient skin compared to healthy control skin. Formalin‐fixed‐paraffin‐embedded (FFPE) 5 mm skin biopsies from FMS patients and healthy, matched control donors were cut into 3 μm sections and stained by immunofluorescence for CD4 (white), CD8 (green), FoxP3 (red) with DAPI nuclear counterstain (blue). Representative staining shown in (a). We assessed the total number of lymphocytes (b) in FMS patients (*n* = 13) and controls (*n* = 10). We also assessed the number of CD8^+^ (c), CD4^+^ (d) and CD4^+^FoxP3^+^ T cells/mm^2^ in FMS patients (*n* = 13) and healthy matched control donors (*n* = 22). Data are represented as mean ± SEM. Statistical significance calculated by unpaired two‐tailed Student's *t*‐test. ns, *p* > 0.05; **p ≤ 0.01.

Given the predisposition of FMS patients to cutaneous melanoma and FMS fibroblasts secreting less inflammatory proteins (Figure 4), T cell numbers in FMS patient skin were enumerated using immunofluorescence (Figure 7a). While there was no significant difference in total lymphocyte count (Figure 7b), the skin of FMS patients exhibited reduced numbers of CD8^+^ and CD4^+^ T cells (Figure 7c,d) when compared to healthy control individuals.

Proportions of FoxP3^+^ CD4^+^ regulatory T cells remained equivalent between both groups (Figure 7e). Reductions were restricted to the lymphocyte compartment; numbers of total CD45^+^ leucocytes, melanocytes, dermal fibroblastic cells, Langerin^+^ Langerhans cells, CD11c^+^ dermal dendritic cells and CD163^+^ macrophages were preserved between patients and controls (data not shown). To understand the mechanisms underlying the decreased T cell numbers we investigated whether peripheral blood T cells from FMS patients had altered capacity to migrate to or be retained in the skin. FMS patient and control skin was stained for tissue retention marker CD69. No differences in expression of CD69 on CD4^+^ and CD8^+^ T cells was seen (Figure S7a,b). We also investigated the expression of the skin homing receptors CLA and CCR4 by flow cytometry. FMS patient peripheral blood CD4^+^ and CD8^+^ T cells expressed CLA and CCR4 comparably to healthy control cells (Figure S7c–f). The similarities in tissue retention marker and skin homing receptor expression suggests the attenuated SASP production from FMS patient fibroblasts in the diminished accumulation of T cells in FMS patient skin.

## 4 DISCUSSION

Studying humans with the germline heterozygous *CDKN2A*‐mutation has provided a unique chance to better understand the nuanced and complex role of p16^INK4a^ in human biology and pathophysiology. Here we have identified multiple cellular senescence associated defects in melanocytes and other cell types in FMS patients in vivo and in isolated cell populations in vitro, that may collectively increase the risk of these patients to the development of melanoma. This work has enabled more detailed analysis of the impact of the p16^INK4a^ mutation at a tissuespecific cellular level. We have shown that the *CDKN2A* mutation leads to reduced p16^INK4a^ expression and that melanocytes are particularly affected. However, there is also lack of ageassociated p16^INK4a^ upregulation in both melanocytes and dermal fibroblastic cells in the patients indicating that other cutaneous cell types in addition to melanocytes are also affected by the *CDKN2A* mutation. The decreased proportions of p16^INK4a^ positive melanocytes together with the reduced numbers of TAF^+^ in these cells indicates decreased melanocyte senescence in the skin of FMS patients compared to healthy age and sex matched controls. The germline *CDKN2A* mutation also confers to patients an increased risk of developing pancreatic cancer (Goldstein et al., 1995; Lynch et al., 2002). The preferential use of p16^INK4a^ senescence pathway by melanocytes may be mirrored by pancreatic exocrine cells. Indeed, approximately 95% of pancreatic ductal adenocarcinomas have functional loss of the *CDKN2A* gene (Rozenblum et al., 1997; Schutte et al., 1997).

The SASP is a hallmark feature of senescent cells, and encompasses the secretion of soluble pro‐inflammatory cytokines, chemokines, growth factors, angiogenic factors and matrix metalloproteinases (Coppé et al., 2008). p16^INK4a^ has a role in SASP secretion, with loss of p16^INK4a^ in oncogene‐induced senescent human fibroblasts and melanoma tumour cells decreasing various SASP factors including *IL‐6* and *CXCL8* expression (Buj et al., 2021). Our study advances on this by showing, in humans, the key observation of senescent FMS patient fibroblasts secreting significantly lower levels of the SASP compared to those from healthy controls. The SASP can recruit immune cells into tissues (Sagiv & Krizhanovsky, 2013) and these cells can recognize and kill senescent and also malignant cells (Iannello et al., 2013; Kale et al., 2020; Kang et al., 2011; Xue et al., 2007). The decreased SASP secretion may therefore hinder immune surveillance that may be a factor that promotes malignancy in these patients.

The importance of T cells in melanoma control has been well‐studied (Brummel et al., 2023; Mahnke et al., 2012; Mukherji, 2013; Muul et al., 1987). It is possible that the reduced secretion of SASP factors may restrict T cell recruitment into the skin of FMS patients that may promote the escape of potentially tumorigenic cells from immune clearance. We have found significantly lower numbers of T cells in the skin of these patients compared to controls that is not due to decreased expression of skin homing or chemokine receptors by peripheral blood T cells or the expression of molecules like CD69 that are responsible for tissue retention of these cells. This suggests that T cells may not be recruited into the skin due to reduced SASP secretion by senescent fibroblasts at the steady state. This possibility requires further investigation.

Fibroblasts from FMS patients have significantly greater replicative capacity than control cells and these can proliferate despite harbouring DNA damage. This extends to a previous report in which melanocytes from patients with mutations in p16^INK4a^ also showed greater replicative capacity compared to healthy controls, however these cells, like fibroblasts from these patients exhibited nuclear aberrations at the point of growth arrest (Sviderskaya et al., 2003). Therefore, both fibroblasts and melanocytes isolated from FMS patients have reduced stringency in the proliferative control of DNA damaged cells. The reason for greater DNA damage‐protein expression in melanocytes compared to fibroblasts is not fully understood. Melanocytes are recognised to be subject to oxidative stress resulting from melanin synthesis (Denat et al., 2014), however defective DNA damage repair mechanisms or other unique aspects of melanocyte biology may also could account for the elevated level of DNA‐damage proteins. Further investigation is required to identify the link between greater levels of DNA damage protein expression in melanocytes and their susceptibility to malignant transformation and development of melanoma rather than the development of fibroblast‐related tumours in these patients.

The *CDKN2A* gene is unique, as during gene expression alternative RNA splicing allows production of four different proteins: p16^INK4a^ and p14^ARF^, both of which are involved in cell cycle regulation albeit through different pathways, as well as p12 and p16𝛾 (Serra & Chetty, 2018). The patients we have investigated in the study have mutations in Exon 1a or Exon 2 with one individual having an intronic mutation upstream of Exon 1a (Figure S1). We did not observe any association between the site of the mutation and the different phenotypic or functional parameters we investigated in this study but do not rule out the possibility that the study of a larger patient cohort may identify some associations.

p16^INK4a^ is a key inhibitor of cellular proliferation makes it crucial for prevention of tumour progression (Serrano, 1997). Mutated p16^INK4a^ is also considered to be a key event in melanoma development, with a majority of melanoma cells lines from sporadic tumours having a p16^INK4a^ mutation (Castellano et al., 1997). The clinical syndrome of a *CDKN2A* mutation resulting in melanomas suggests that melanocytes may be more reliant of the p16^INK4a^/Rb pathway than other cutaneous cell types to become senescent. The germline *CDKN2A* mutation also confers to patients an increased risk of developing pancreatic cancer (Goldstein et al., 1995; Lynch et al., 2002).

In summary we have demonstrated that the loss of p16^INK4a^ function in FMS patients has a significant impact on the senescence features of melanocytes and fibroblasts in vivo and in vitro. The study of these patients has provided insight on how senescence is regulated in multiple cell types within a single organ and has identified possible ways by which the loss of proliferative control may lead to the development of malignancy. These studies are also highly relevant for understanding the role of senescent cells in regulating immunity in tissues during ageing (Chambers et al., 2021; Pereira et al., 2019) and to understand the impact of senolytic drugs on different senescent cell types in vivo (Bracken et al., 2024).

## AUTHOR CONTRIBUTIONS

PS designed and performed experiments and wrote the manuscript. PL and SS performed experiments and contributed to manuscript writing. OPD and ESC provided experimental support, CHF assisted with statistical analyses. SNK and AK provided access to and recruitment of patients, DCOG was involved in guidance for the direction of the project, MHAR supported overall project design. KEL provided intellectual guidance, contribution to study design and clinical support. ANA obtained funding, designed experiments, coordinated collaborative efforts between researchers contributed to writing and editing the manuscript.

## CONFLICT OF INTEREST STATEMENT

The authors declare no conflict of interest.

## Supporting information


Data S1.

## Data Availability

The data that support the findings of this study are available from the corresponding author upon request.
