# Correction to “Multiple Outcomes of the Germline p16^INK4a^ Mutation Affecting Senescence and Immunity in Human Skin”

**DOI:** 10.1111/acel.70157

**Published:** 2025-06-26

Subramanian, P., Sayegh, S., Laphanuwat, P., Devine, O. P., Fantecelle, C. H., Sikora, J., Chambers, E. S., Karagiannis, S. N., Gomes, D. C. O., Kulkarni, A., Rustin, M. H. A., Lacy, K. E., & Akbar, A. N. (2025). Multiple outcomes of the germline p16^INK4a^ mutation affecting senescence and immunity in human skin. *Aging Cell*, 24, e14373.

In the “Acknowledgements” section the text “We thank Hugh Trahair for his technical support with sectioning skin blocks. We also thank Dr. Rajni Sharma helping to set up the in vitro senescence assay” is incomplete. This should have read “We thank Hugh Trahair for his technical support with sectioning skin blocks, Dr Zena Willsmore for obtaining donor information, and Histopathology colleagues at Guy's and St Thomas' Hospital for their support with sectioning patient blocks. We also thank Dr. Rajni Sharma for helping to set up the in vitro senescence assay.”

We apologize for this error.

