# Supplementary material for: Multiple outcomes of the germline p16INK4a mutation affecting senescence and immunity in human skin
Source: Aging Cell. 2024 Oct 17;24(2):e14373. doi: 10.1111/acel.14373 (PMC11822638; doi:10.1111/acel.14373)

Supplementary Table 1

Patient	Number of Melanomas	Age at 1st Melanoma	Mutated Gene Element	Nucleotide Change
1	1	19	Information not available	
2	0	NA	Exon 1a	c.9_32dup
3	3	33	Exon 2	c.383_387delinsGATGCG
4	3	38	Exon 1a	C.104 G>C
5	8	31	Exon 2	c.159 G>C/c.202G>C
6	0	NA	Exon 2	c.159 G>C.
7	2	21	Exon 1a	c.146T>G
8	1	24	Exon 2	c.159 G>C.
9	1	49	Exon 1a	c.113C>G
10	1	18	Information not available	
11	1	36	Exon 1a	C.104 G>C
12	0	NA	Exon 1a	c.71G>C
13	0	NA	Exon 2	c.159 G>C.
14	0	NA	Exon 2	c.206A>G
15	1	47	Exon 2	c.159 G>C.
16	1	24	5'UTR	c.-34G>T

Supplementary figure 1

(A)

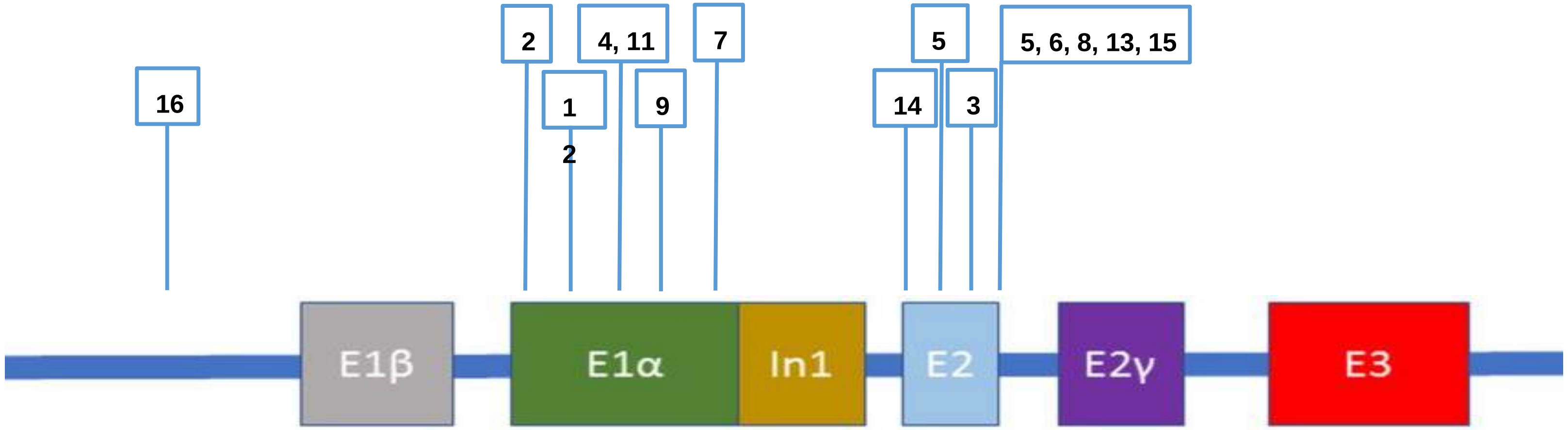

(B)

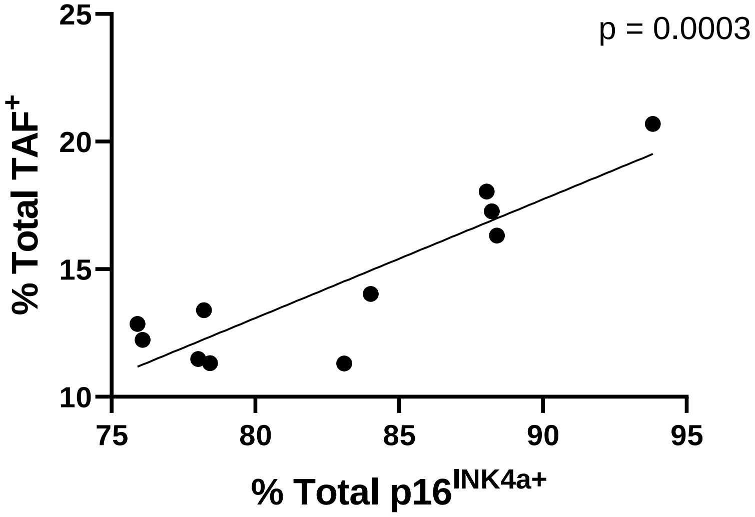

Supplementary figure 2

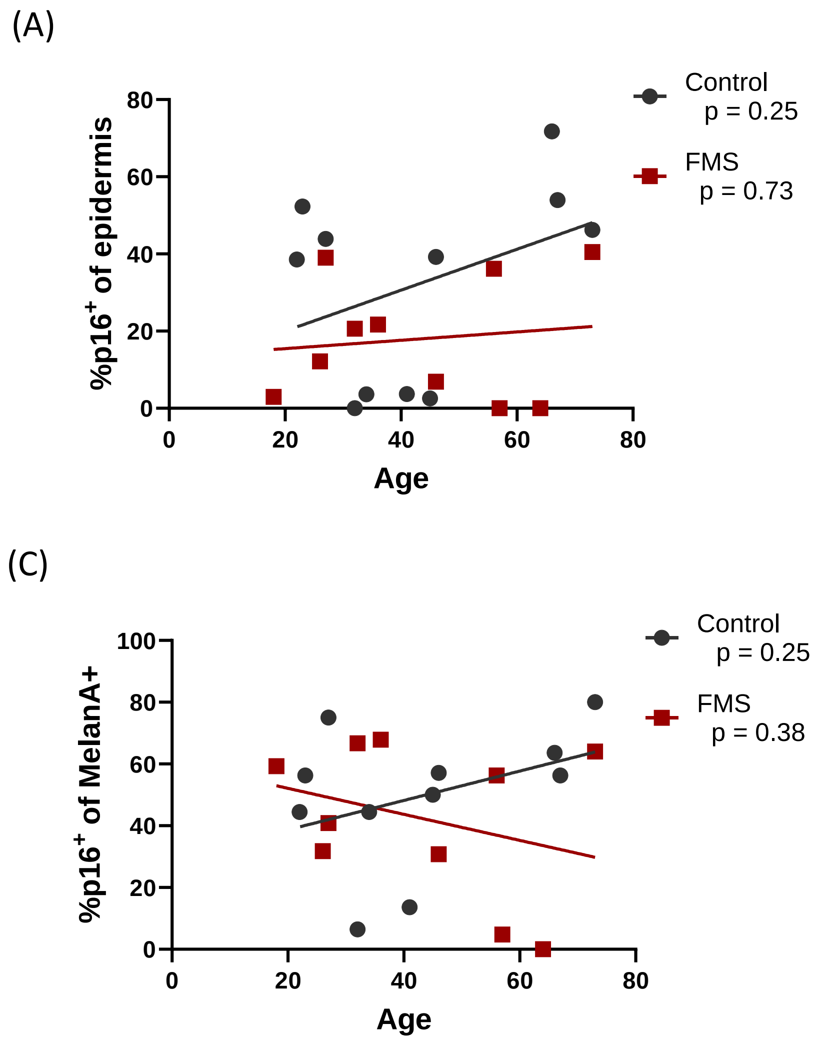

Supplementary figure 3

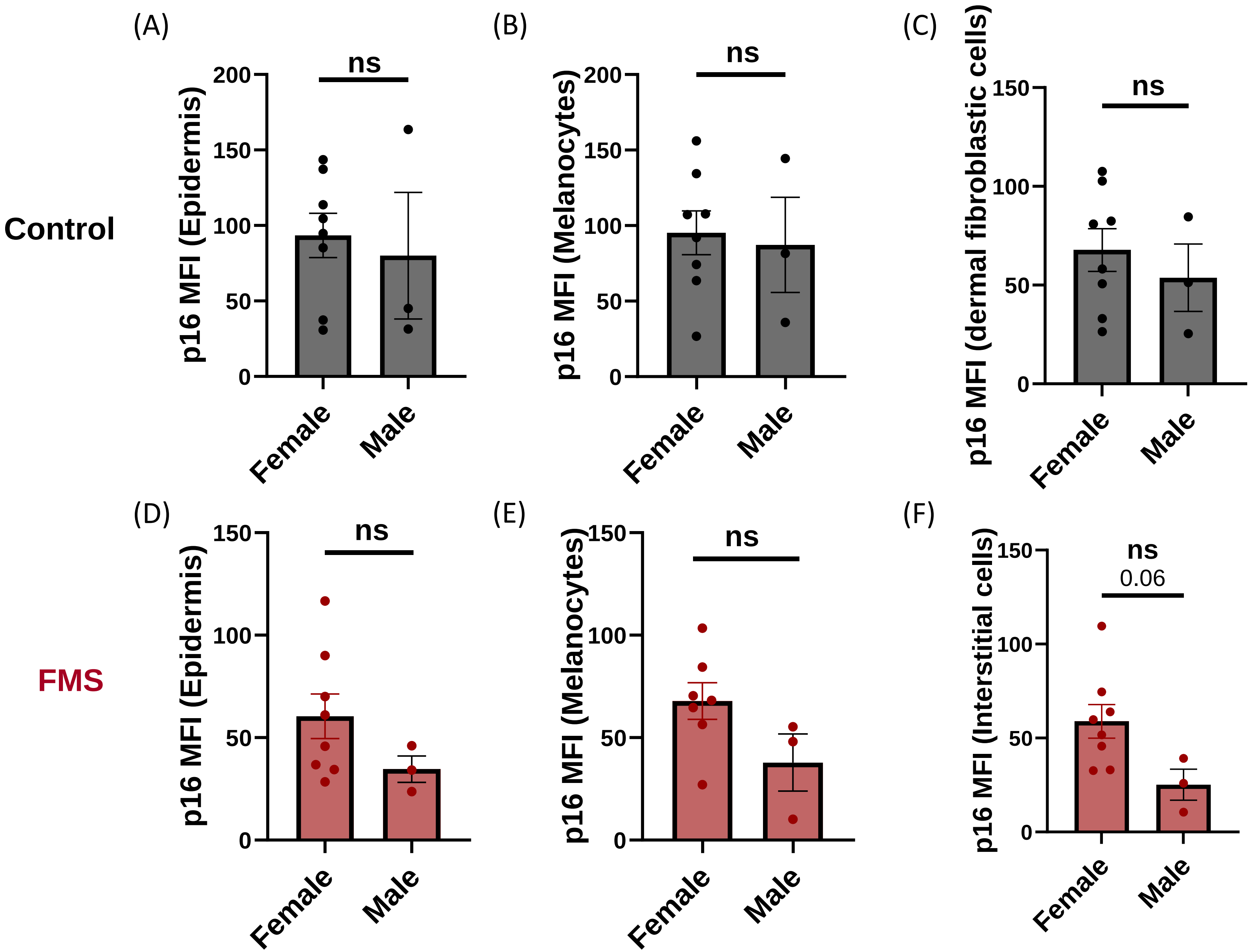

Supplementary figure 4

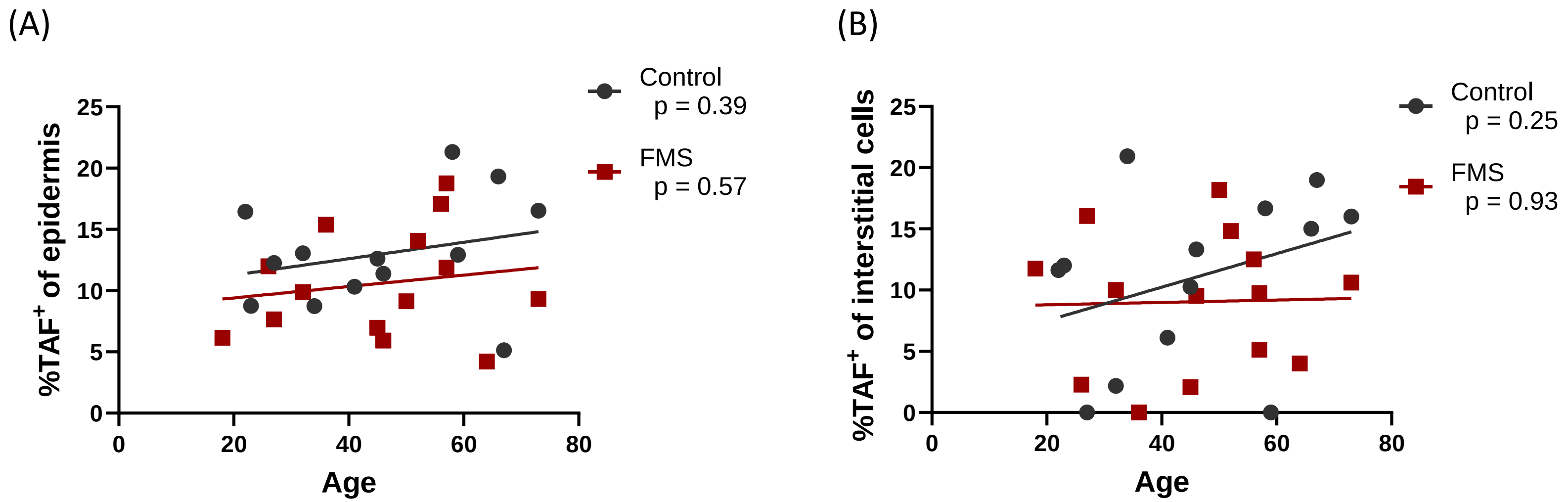

Supplementary figure 5

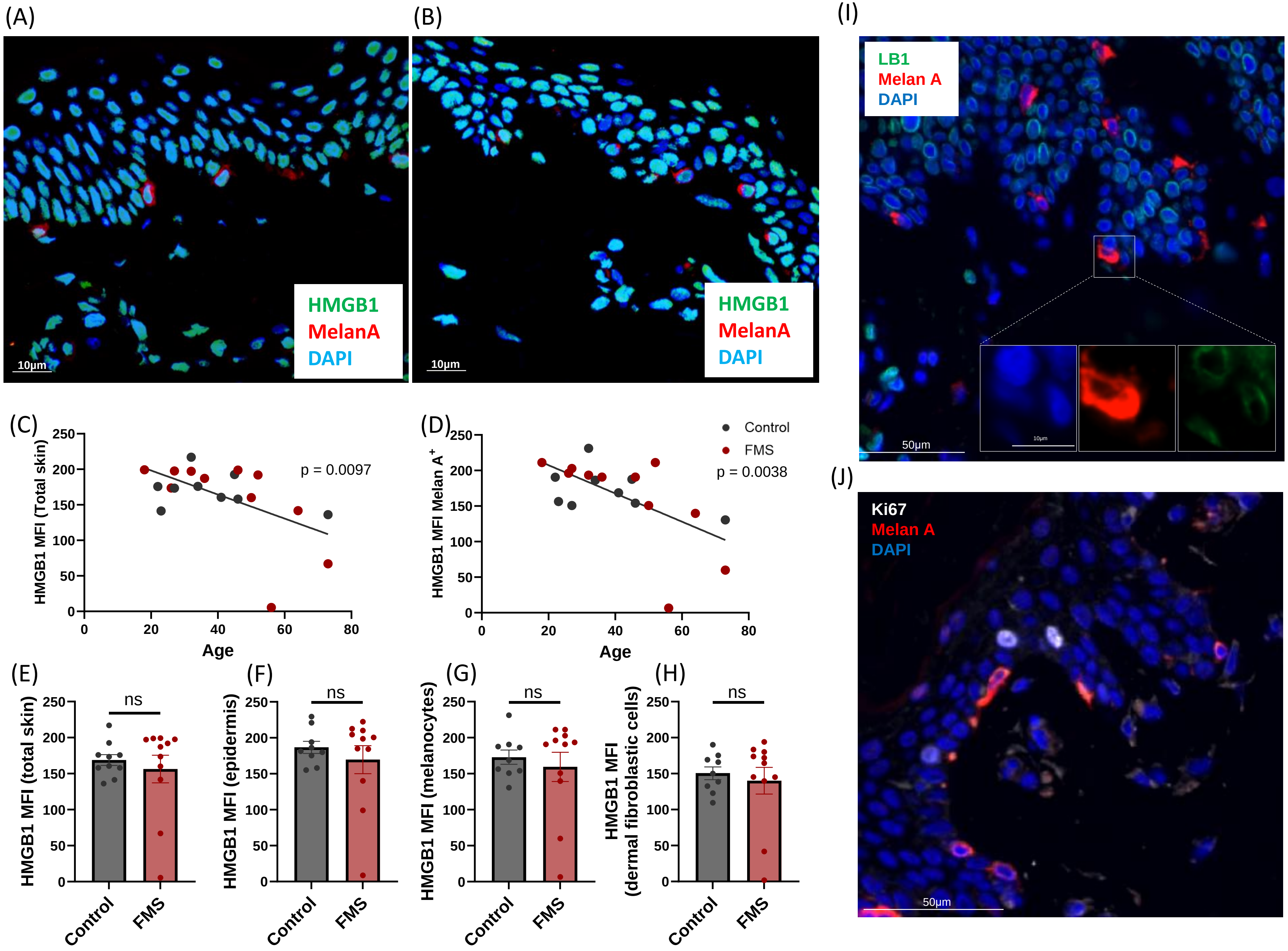

Supplementary figure 6

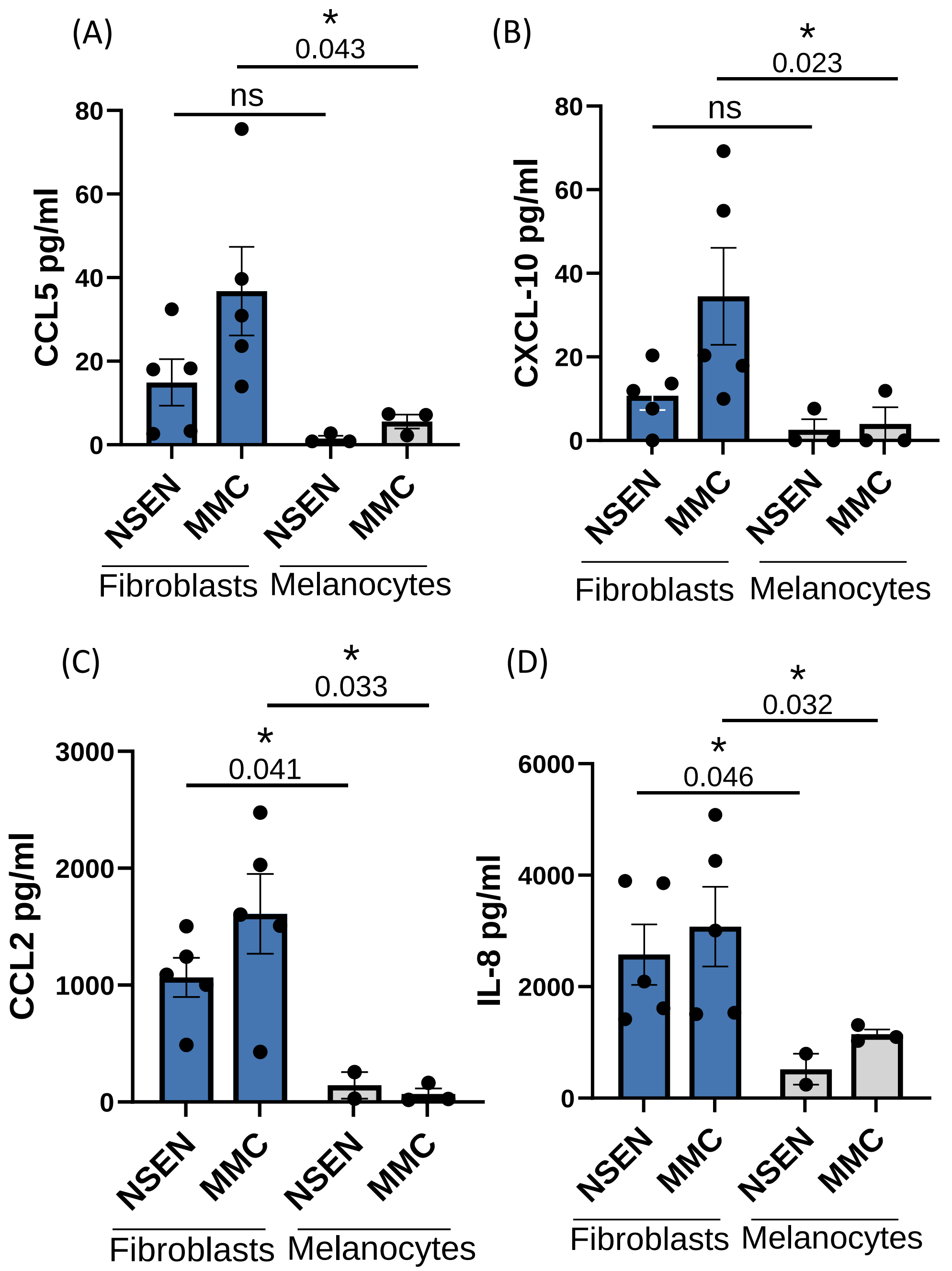

Supplementary figure 7

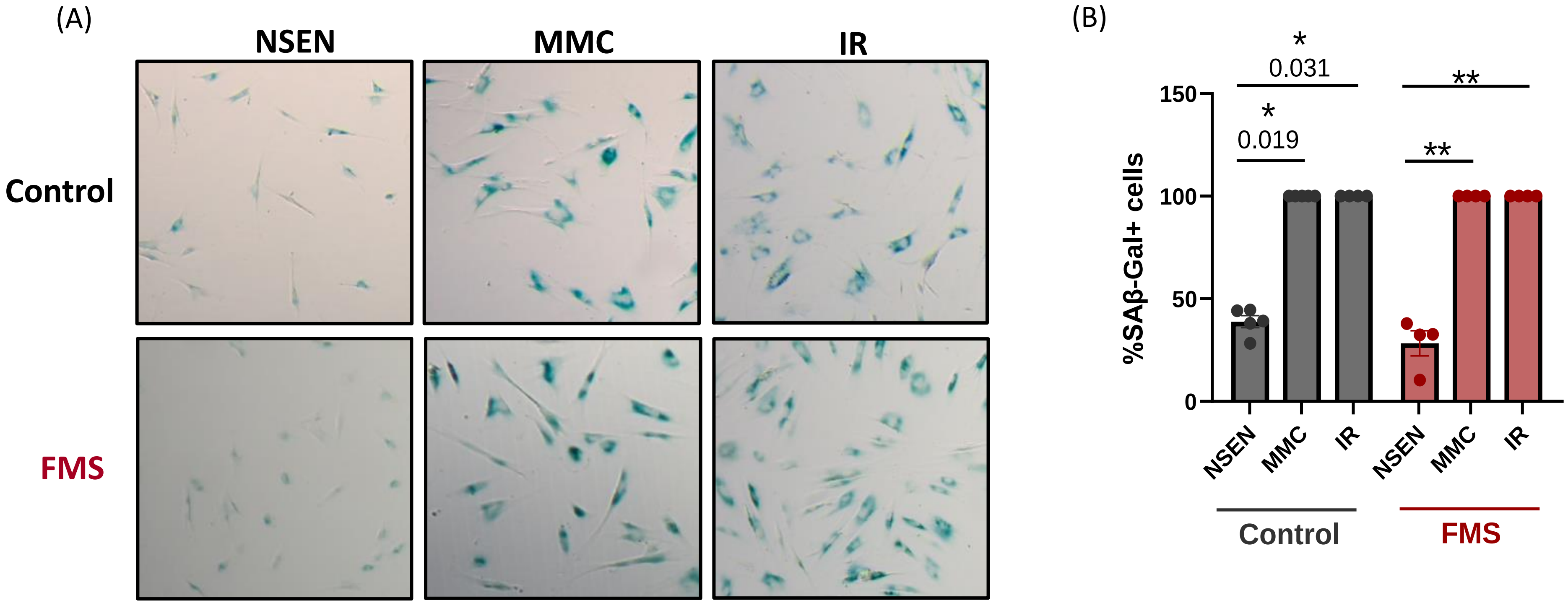

Supplementary figure 8

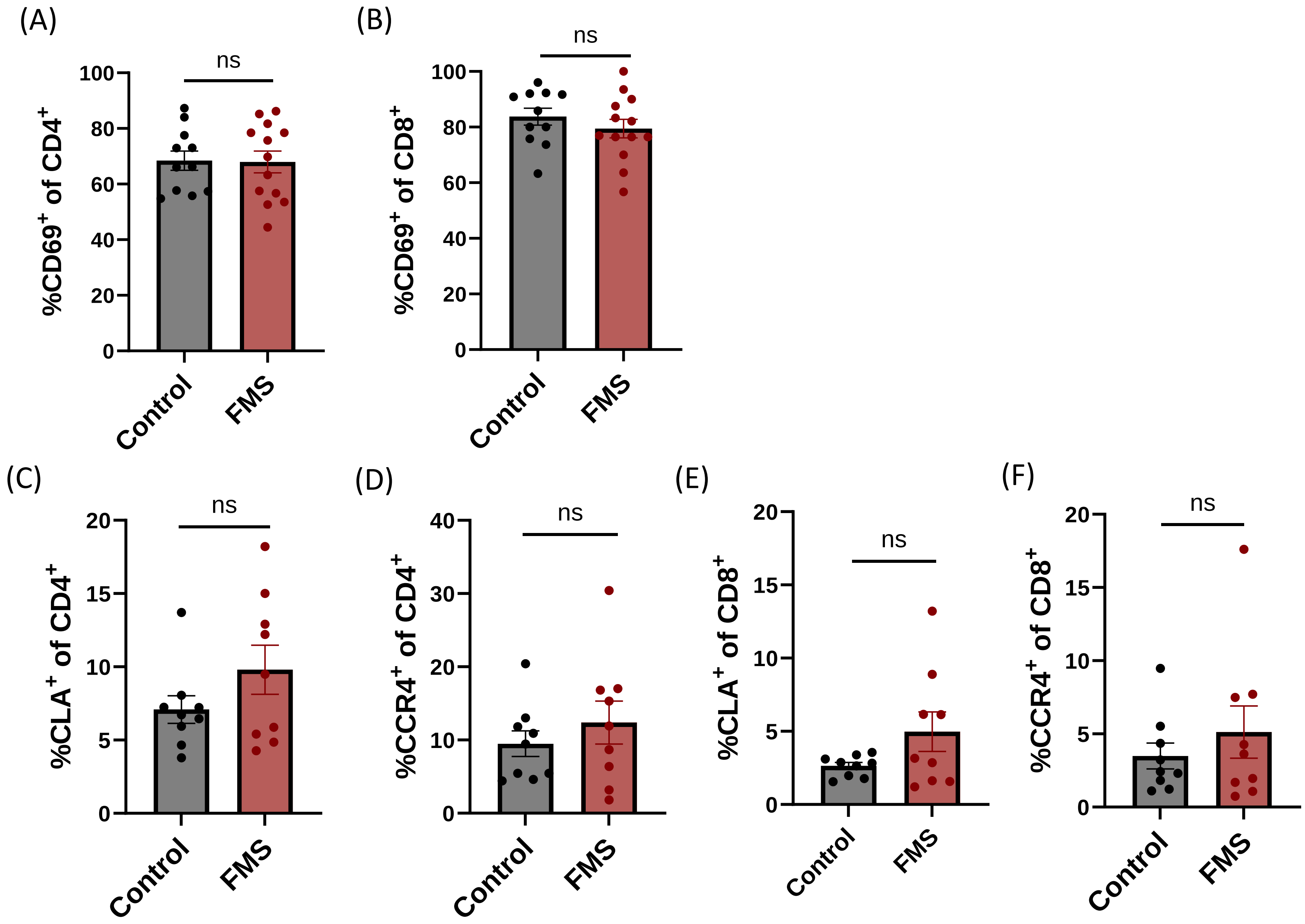

Supplement: Supplementary file 1 — Data S1. [file ACEL-24-e14373-s001.pdf]
